# Promotion of Healthy Lifestyles to Teenagers with Mobile Devices: A Case Study in Portugal

**DOI:** 10.3390/healthcare8030315

**Published:** 2020-09-02

**Authors:** María Vanessa Villasana, Ivan Miguel Pires, Juliana Sá, Nuno M. Garcia, Maria Canavarro Teixeira, Eftim Zdravevski, Ivan Chorbev, Petre Lameski

**Affiliations:** 1 Faculty of Health Sciences, Universidade da Beira Interior, 6200-506 Covilhã, Portugal; julianasa@fcsaude.ubi.pt; 2 Instituto de Telecomunicações, Universidade da Beira Interior, 6200-001 Covilhã, Portugal; impires@it.ubi.pt (I.M.P.); ngarcia@di.ubi.pt (N.M.G.); 3 Computer Science Department, Polytechnic Institute of Viseu, 3504-510 Viseu, Portugal; 4 Cova da Beira Hospital Center, 6200-251 Covilhã, Portugal; 5 UTC de Recursos Naturais e Desenvolvimento Sustentável, Polytechnique Institute of Castelo Branco, 6000-909 Castelo Branco, Portugal; ccanavarro@ipcb.pt; 6 CERNAS—IPCB, Research Centre for Natural Resources, Environment and Society, Polytechnique Institute of Castelo Branco, 6000-909 Castelo Branco, Portugal; 7 Faculty of Computer Science and Engineering, University Ss Cyril and Methodius, 1000 Skopje, North Macedonia; eftim.zdravevski@finki.ukim.mk (E.Z.); ivan.chorbev@finki.ukim.mk (I.C.); petre.lameski@finki.ukim.mk (P.L.)

**Keywords:** teenagers, mobile application, nutrition, physical activity, health, education

## Abstract

Educating teenagers about nutrition and promoting active lifestyles is essential in reducing the long-term health risks and one idea to achieve this is by using mobile applications. Previous studies showed that the existing mobile applications have similar functionalities, such as intervention with questionnaires, and the use of gamification techniques to improve interactiveness. However, unlike our study, some studies are not validated and verified by healthcare professionals. Additionally, this study intends to promote the interaction between the teenagers and the medical communities. In this study, we analyze the benefits of the proposed mobile application, which features monitoring of physical activity, daily tips and curiosities, questionnaires, and gamification through earning points. Most of the teenagers were satisfied with the physical activity monitoring and found the tips, curiosities, and weekly questionnaires useful. The study started with 26 teenagers from two schools in the center of Portugal that would use the mobile application for five weeks. Still, at the end of the study, only 7 teenagers finalized the study. The decreasing number of teenagers in the study was affected by the lack of social interaction caused by the pandemic situation. During the period, the mobile application would engage the users with notifications on nutrition and physical activity, challenges concerning the number of steps and calories they would have to spend, and questionnaires related to the curiosities and suggestions from the previous week. We used Fisher’s test to investigate the relationship between the assessment obtained in the responses to the questionnaires, and the adoption of healthier eating and sports practices. In summary, participants were satisfied with the mobile application and experienced some improvements in diet and habits.

## 1. Introduction

Inadequate knowledge about nutrition and physical activity causes bad habits in young people. Teenagers are spending a lot of hours in sedentary activities, practicing a low level of physical activity [1,2]. Consequently, one of the most significant health problems related to the teenagers is the obesity [3,4,5]. Studies identified a relationship between teenagers’ poor habits to socioeconomic factors [6] and energy intake [5].

Obesity is defined as a chronic, complex, and multifactorial disease that is unfavorable for health. It is characterized by an excessive increase in body fat, resulting from the imbalance of caloric expenditure and energy intake [7]. This imbalance is favorable to the development of several metabolic complications, namely insulin resistance, which leads to hyperglycemia, dyslipidemia, hypertriglyceridemia, low levels of high-density lipoprotein, and hypertension [8]. The best way to spend calories is through physical activity, which better influences energy balance and weight control [9].

The reduction and control of the incidence and prevalence of overweight and obesity in the child and school population is one of the goals proposed for 2020 in the National Health Plan—Review and Extension to 2020 [10]. Thus, in Portugal, the General Directorate of Health, created the National Program for the Promotion of Healthy Eating, in which public health strategies for combating obesity are addressed and created. Since 2017, information on the levels of physical activity and physical inactivity of users of the National Health Service has been gathered [11].

For children, adolescents and young adults aged between 5 and 19 years, the World Health Organization (WHO) defines excess weight as the Body Mass Index (BMI) for age with more than a typical deviation above the median established in child growth patterns and obesity as being higher than two standard deviations above the norm established in child growth patterns [7]. Thus, children, adolescents, and young adults aged 13 to 19 years between the 85th and 95th percentile are overweight and above the 95th percentile are classified as obese children [12]. In recent years, there has been an increase in obesity among children, adolescents, and young adults in many countries, and its fight has been the target of several measures in the field of public health [13]. According to the WHO, in 2016, the number of children, adolescents, and young adults (5–19 years), where overweight or obese teenagers exceeded 340 million [7].

As an attempt to tackle this obesity problem, the aim of this study consists in using a mobile application for the promotion of healthy physical activity and nutrition habits to teenagers. The target population needs several mechanisms to stimulate physical activity, including gamification, medical control, and personalized advices. In particular, with the gamification approach, we attempt to apply typical elements of game playing (e.g., point scoring, competition with others, rules of play) to the healthy nutrition and physical activity, to encourage engagement with the mobile application.

The scope of this project includes teenagers from two public schools of the municipalities of Fundão and Covilhã (center of Portugal), where the teenagers used a mobile application for five weeks with the different functionalities proposed at [14]. After this time, the teenagers answered a questionnaire related to satisfaction with the use of the mobile application. This paper analyses the evolution of the physical activity and nutrition habits of the teenagers involved in the study with a mobile application.

This remainder of the article is structured as follows. Section 2 presents the related work based on the analysis of the world’s obesity, mobile applications related to nutrition and physical activity available in the Google Play store, and studies with similar our project methodology with teenagers. The methods implemented are presented in Section 3, showing the results of this study in Section 4. This paper reports the discussion in Section 5, concluding this paper with the conclusions in Section 6.

## 2. Related Work

The study [15], published in 2018, presents data collected between 2015 and 2016 in Portugal, concluding that the prevalence of obesity increases with increasing age, being less prevalent in children and higher in the elderly. There are three critical periods in life-related to the prevalence of obesity, namely at 5 years old, 15 years old, and finally, 75 years old. This study revealed that approximately 17.3% of children (aged less than 10 years old) have pre-obesity and 23.6% of adolescents, aged between 10 and 18 have pre-obesity (i.e., overweight). Also, 7.7% of children, as well as 8.7% of adolescents, were obese [16,17].

According to studies carried out in Europe, adolescents in Europe consume high amounts of fast food and sugary drinks, and spend less time on family meals compared to previous generations [18]. In adolescence, healthy eating is being put aside, decreasing the consumption of fruits and vegetables, and increasing the consumption of sweets and soft drinks. They consume half the recommended amount of fruits and vegetables and less than two-thirds of the recommended amount of milk and dairy products, consuming more meat, fats and sweets than recommended [18].

Regarding physical activity among young people in Europe, the levels are generally deficient, being even lower among young women and decreasing as they progress through adolescence [9], sedentary behaviors dominated the daily lives of adolescents [19], reaching 60% of the regular time of young people spent in this type of activities [20], about nine waking hours [18]. Around 11 and 13 years old, there is a more marked increase in this type of behavior [21].

One of the significant problems of childhood obesity is reflected by data on the prevalence of physical activity in Portugal, collected between 2015 and 2017, which estimate that only about half of children reach WHO recommendations [11]. Some data suggest that recommendations for physical activity are not met in three-quarters of the Portuguese population over 15 years old [22].

A study carried out in 2017 by the WHO [23], estimated that as the adolescents are older, the level of physical activity decreases, based on analyzing the answers to the question regarding doing “at least one hour of moderate to vigorous activity every day”, it was answered positively by 25% of children aged with 11 years old. Still, for those aged with 15 years old, the number drops to 16%. In this study, we also conclude that the older adolescents are more likely to have sedentary behaviors. Other studies support this and also suggest that even more complex patterns are present [24]. With children aged with 11 years old, only 50% report watching two or more hours of television during the week, against 63% of those who are older [23]. The study [9] reports the converse, registering a decrease in obesity in individuals aged with 11, 13, and 15 years old with increasing age.

Other works address a similar problem, but about physical inactivity of adults in Sweden, such as [25]. The design of the study is similar, and also similarly to our work, where we monitor the participants for 5 weeks, in this study, participants were monitored for 4 weeks.

Based on the research of the strategies implemented to encourage the use of this type of mobile applications by young people, we found that it is necessary to innovate regularly with different ideas, requiring an interactive design, ease of use, frequent updating of information, use of questionnaires as interaction with young people, use of gamification and promoting benefits from using the application [26,27,28,29,30].

## 3. Methods

### 3.1. Overview

To understand which methodologies are used worldwide to captivate young people to have healthy living habits, and educate them using mobile applications, a review of the studies available in the literature was performed and published in [31], using the Natural Language Processing (NLP)-based framework for an automatic search in digital libraries and identifying relevant studies [32]. It was verified that all the analyzed studies use questionnaires as an evaluation method, mainly promoted using mobile applications to promote healthy habits, including nutrition and physical activity habits.

Given that mobile applications are used to control lifestyle habits [33,34], and that young people use mobile devices [35] and mobile applications [36] frequently, a systematic review was carried out to study which mobile applications are most used by people who use devices with the Android operating system and which are related to diet, nutrition, health, and physical activity [37]. Mostly, the mobile applications are related to diet and nutrition, and the features that were most present were weight, height, age, sex, goals, calculation of necessary calories, diet diary, food database and its calories, calories burned, and the calculation of calorie intake.

### 3.2. Study Design and Participants

A mobile application was proposed in [14]. The application was built with the aim of monitoring, advising and educating young people about health. In this freely available mobile application, CoviHealth (“Covilhã” + “Health”), young people could register their diet, physical activity, and medication plans, and add and alter their anthropometric data, alerts, and objectives. They could also accept the challenges that were launched and answer the questionnaires regarding the information given in the previous week, through curiosities and offered suggestions. These curiosities and suggestions were related to nutrition and physical activity and their effects on health.

Also, CoviHealth application performs the monitoring of physical activity with the pedometer and measurement of the calories. The teenager could also use the possibility to fill his/her training and diet plans.

**Figure 1:**
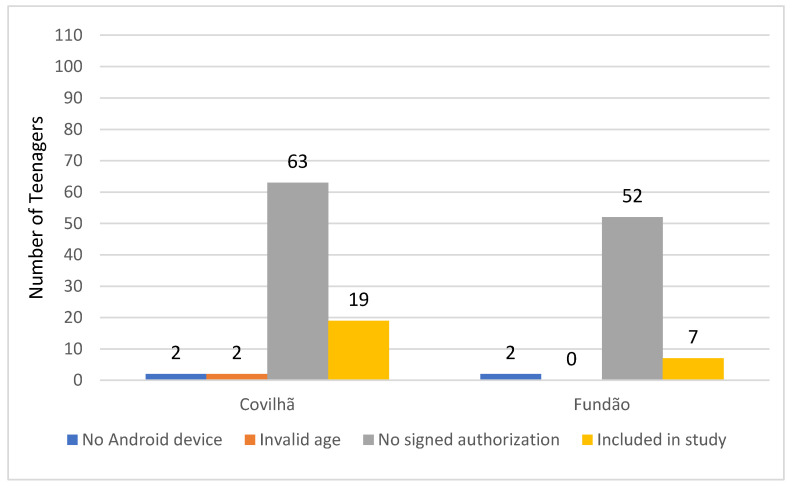
Selection process of the participants in the study from two schools.

Figure 1 presents the selection of the participants in the study using different criteria. The criteria was that participants must possess a smartphone with Android operating system, to be aged between 13 and 18 years old, must have a signed authorization from parents with a provided valid email address, download the mobile application, and fill in the initial questionnaire with correct information [38]. The reason why only a subset of participants from the initial eligible population was included in the study is because their parents did not provide a signed authorization providing consent for their children to participate in the study. In practice, there were many other downloads of the mobile application from the Play Store as we could not limit this, but that data was completely discarded from the study due to the selection process described above. The distribution of the population of this study was gender-balanced, starting 26 young people (i.e., 14 are female, and 12 are male) with a height between 1.43 m and 1.87 m, weight between 35 kg and 80 kg, and BMI between 15.10 and 28.52. Still, in the end, only seven students participated (i.e., 3 are female, and 4 are male). The remaining teenagers were aged between 13 and 16 years old with a height between 1.43 m and 1.71 m, weight between 35 kg and 75.6 kg, and BMI between 15.10 and 25.90. The decreasing number of teenagers was related to the pandemic situation during the implementation of the study. The study was implemented between 21 February 2020 and 27 March 2020. Thus, the mobile application was only distributed for a closed group of teenagers in alpha testing mode by Google Play Store.

These young people used the mobile application for five weeks, during which 18 curiosities and 10 suggestions on nutrition and physical activity were provided as well as six challenges about the number of steps and calories they would have to spend. The mobile application was also used for the distribution of four questionnaires related to the tips and curiosities available [38].

According to studies in the literature [39,40], five weeks could be enough to change some personal habits and the process can be done in such a short time due to the changes of environment which is helped by smartphones and other electronic devices.

At the end of the four weeks, young people answered a satisfaction questionnaire available about the mobile application and its different functionalities [38]. The questionnaire also evaluated the interest in other features that are not yet available, including the possibility of each young person to be followed and advised by their healthcare professional.

Fisher’s non-parametric test was used to study the independence between the variables under study, as an alternative to the Chi-square test because the sample is small [41]. SPSS Statistics software (IBM, New York, NY, USA) [42] was used to perform statistical analyzes with a significance of 5%.

### 3.3. Ethics Statement

The research was previously approved with code CE-UBI-Pj-2019-028 by the Ethics Commission of Universidade da Beira Interior, Covilhã, Portugal. The research was conducted with a mobile application that controlled its use with authentication of the different teenagers. The transfer of text between the mobile application and the server was encrypted, using the Security Sockets Layer (SSL) protocol. The parents authorized the use and download of the mobile application by the teenagers. The access to the mobile application was performed with a username that does not identify the teenager. Thus, all actions were performed anonymously and with confidentiality. In the group challenges, the different participants did not know each-other. Finally, the teenagers or their parents could ask for the removal of the data from the database at any time.

## 4. Results

### 4.1. Results of the Participation in the Study

**Figure 2:**
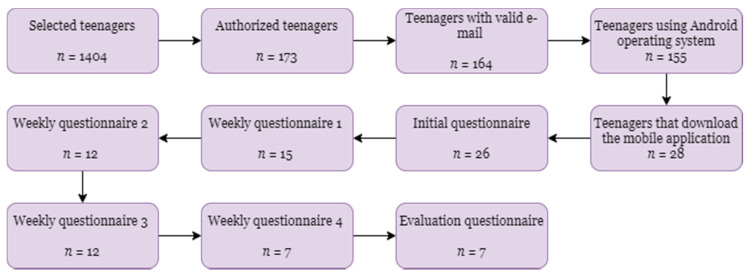
Evolution of the number of teenagers during the study.

At the beginning of the study, as presented in Figure 2, 1404 teenagers from two schools placed in Fundão and Covilhã (Portugal) municipalities were selected. Of those selected, only 173 teenagers were authorized by the parents to participate in the study. Next, only 164 teenagers provided a valid email to send the invitation for the participation in the study. Of those with valid emails, only 155 teenagers reported that they have a smartphone with Android operating system. Between them, only 28 students downloaded and installed the mobile application.

The study started on 21 February 2020, and 26 teenagers filled in the initial questionnaire. At that moment, the dispersion of the pandemic situation increased, and the teenagers could not be in physical contact with others. Thus, at the first weekly questionnaire, only 15 teenagers answered the questions. In comparison, weekly questionnaire 2 reduced the number of answers to 12 teenagers. This number maintained between the weekly questionnaire 2 and the weekly questionnaire 3. Unfortunately, the pandemic situation had a very negative impact, and the number of answers reduced to 7 teenagers in the weekly questionnaire 4 and the evaluation questionnaire.

### 4.2. Sample Analysis

**Figure 3:**
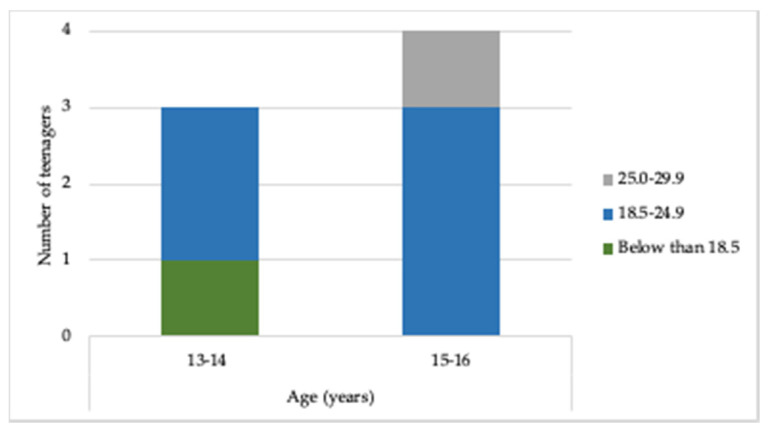
Distribution of students by age and Body Mass Index (BMI).

Figure 3 presents the age and BMI distributions. The 16-years-old teenagers are the most present in the study. On the other hand, the average age of the participants is 15-years-old, given the low variability. Between the teenagers aged 13–14 years, 2 teenagers have BMI between 18.5 and 24.9, and 1 teenager has BMI below 18.5. Between the teenagers aged 15–16 years, 3 teenagers have BMI between 18.5 and 24.9, and 1 teenager has BMI between 25.0 and 29.9.

### 4.3. Analysis of Population Habits

This subsection describes the student’s habits in relation to physical activities, based on the data of all 26 participants in the study. This information was collected at the beginning of the study. Regarding sports, 71.4% of young people practice sport, but only 43% of young people go to the gymnasium, sports complexes, or swimming pools. Following the frequency of exercising, 42.9% of the teenagers are practicing more than three times per week, and 57.1% are practicing less or equals to 3 times per week. Next, following the analysis of the time of exercise, 14.3% of the teenagers are practicing less than 1 h per session, and the remaining 85.7% of the teenagers are practicing more than 1 h per session.

**Figure 4:**
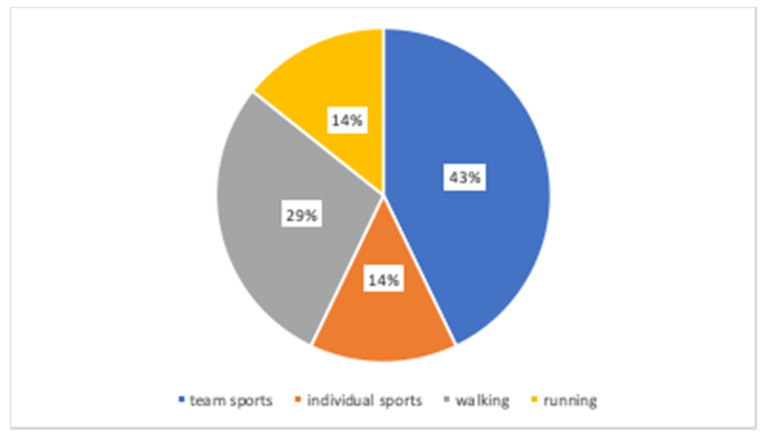
Distribution of students by the preference of physical activities.

Figure 4 shows the preference for physical activity of the different teenagers, verifying that 42.9% prefers team sports.

Regarding diet, 85.7% of teenagers had no specific diet, where the remaining 14.3% were macrobiotic. Next, 57.1% of teenagers consumed one to two servings of fruit and vegetables per day, and the other 42.9% consumed between three and five servings per day.

Regarding the consumption of sweets, savory snacks, or soft drinks, 57.1% of teenagers said they consume less than three times per week, and the remaining 42.9% consume three or more times per week.

### 4.4. Analysis of Weekly Questionnaires

For the analysis of weekly questionnaires, each correct answer gave 1 point to the teenagers. Each questionnaire has four questions related to the tips and curiosities shared by the mobile application about nutrition and physical activity during the last week. The level of difficulty of the questions was maintained during the study. Table 1 shows that the average number of correctly answered questions was higher in weekly questionnaire 3, and the number of correctly answered questions in the first and last questionnaires was the same.

### 4.5. Results of Feedback

As previously mentioned, at the end of the 5-week period, a satisfaction questionnaire was conducted. Following the results obtained with the satisfaction with the physical monitoring, the students were mainly satisfied, except for one student (14.3%).

After the five weeks of the study, the final questionnaire evaluated the physical activity level of the teenager and 4 of the students (57.1%) kept the level of physical activity on similar level as before the study, and the remaining three students (42.9%) increased it. At the same time, 85.7% of the students were satisfied with the training plan, and only one student (14.3%) was not satisfied. Regarding diet, 5 of the students (71.4%) kept their habits, and the remaining 28.6% claimed that their dietary habits improved.

In respect of the availability of tips and curiosities, 28.6% of the students kept their nutrition and physical activity habits, but the remaining 71.4% improved it. Furthermore, 57.1% considered the tips and curiosities useful. Regarding the weekly questionnaires, 71.4% found it helpful to consolidate the knowledge with tips and curiosities. Regarding the medical follow-up, 71.4% considered it important.

The use of gamification and challenges motivated 71.4% of the participants. The same number of persons that are satisfied with the mobile application said that they would use it in the future (85.7%).

### 4.6. Global Results of the Mobile Application

Initially, it was found that the inclusion of advertising in the project increases adherence to this type of mobile applications by young people, and the sessions held in selected schools increased the acceptance of the mobile application. Regarding the students that participated in a presentation about the project, i.e., students from Covilhã (Portugal), 23% downloaded the mobile application. Next, related to the students from Fundão (Portugal), where no presentation was made, only 11% downloaded it.

The young people are aged between 13 and 14 years old, and 15 to 16 years. Thus, 42.9% of the young people are aged between 13 and 14 years old, and 57.1% of them are aged between 15 and 16 years old.

For further analysis of the answers related to the consumption of fruit and vegetables, and sweets, savory snacks, and soft drinks, Table 2 established different points associated with the various answers given. Consequently, Table 3 shows the ranking of points obtained by different teenagers.

During the study we discovered the existence of teenagers with bad habits that were able to improve. We correlated the different points obtained with the improvement of the teenagers’ diet and found out that two teenagers (28.6%) with bad habits improved as presented in Table 4. However, we cannot accept that there is a dependency between changing the diet and the points obtained ($\mathrm{Pr}\left(FI\left(X\right)\geq fi\left(x\right)|H_{0}\right)=0.526>0.05)$. However, most of the teenagers kept the diet.

Table 5 shows the relation between the improvement of final physical activity level with the habits of the teenager. Three teenagers started to perform more physical exercises, and 2 of them improved their frequency. We can confirm that the improvement in the level of physical activity and the frequency of physical activity per week is independent ($\mathrm{Pr}\left(FI\left(X\right)\geq fi\left(x\right)|H_{0}\right)=0.486>0.05).$

As for improving the level of physical activity when compared to the relationship with the duration of each training session, it is possible to conclude that there is no relation between them ($\mathrm{Pr}\left(FI\left(X\right)\geq fi\left(x\right)|H_{0}\right)=1>0.05).$ Thus, 6 of the 7 teenagers started practicing more than 1 h of physical exercise, and the level of physical activity of all of them has improved or maintained.

In general, teenagers of different ages are satisfied with the mobile application, as presented in Table 6. We observed that 6 out of 7 teenagers were satisfied with the mobile application, and we can conclude that their degree of satisfaction is independent of their age ($\mathrm{Pr}\left(FI\left(X\right)\geq fi\left(x\right)|H_{0}\right)=1>0.05).$

Finally, based on Table 7, we verified that the teenagers aged between 13 and 14 were motivated by the mobile application gamification feature. Still, only 50% of the teenagers aged 15 and 16 were motivated by the mobile application gamification feature. However, it was not possible to conclude that the level of motivation depends on the age of teenagers, specialty between 13 and 16 years old ($\mathrm{Pr}\left(FI\left(X\right)\geq fi\left(x\right)|H_{0}\right)=0.429>0.05).$

## 5. Discussion

Other studies have been performed with teenagers and the use of mobile applications. Considering the different studies found in the literature, Table 8 shows a comparison of the studies previously analyzed, verifying that three studies revealed a percentage of abandonment lower than the CoviHealth project.

The authors of [43] verified a reported improvement of the diet. However, the study [44] did not reveal alterations in the diet of the teenagers. In [48], the satisfaction was moderate in comparison of the studies with similar methodology. Next, in [45], the teenagers were satisfied. In conclusion, in [44], the satisfaction was low. However, compared with our mobile application, most teenagers were confident with the use of mobile applications. Thus, as in the study [46], the majority of the users of our project would continue with the use of the mobile application.

Some experts do not recommend the use of mobile devices until the age of 14 years old, other experts until the age of 16 years old [49]. Despite that, this study involves the use of smartphones by 13-year-old adolescents. We do not disagree with these studies, but still, it is a reality that most teenagers, starting from 13 years old or even earlier, use mobile phones. We want to leverage the fact that they have smartphones and put them into good use for obtaining healthy habits.

The healthcare professional has access to all data entered by the teenagers in the mobile application, which allows for close control of the teenagers. Subsequently, data may be validated by the healthcare professional in consultation. All data relating to each teenager can only be entered by him/her or his/her doctor. The measurement of energy expenditure and the pedometer were developed with validated methods [50,51,52,53].

The study proved that the use of a mobile application simulates the teenagers to have good physical activity and nutrition habits. However, this study has some limitations that may affected the results, such as a low number of teenagers that completed in the study, and the mobile application is only focused in physical activity and nutrition. The teenagers are a special population that are usually captivated by the use of these technologies.

## 6. Conclusions

The mobile application named CoviHealth was mainly devoted to the education of teenagers about nutrition and physical activity using dynamic tips and curiosities, gamification, challenges, and the possibility to earn points.

The study started with 26 teenagers, but some of the involved students abandoned the study, finalizing the study with the analysis of the participation of seven students. These students are aged between 13 and 16 years old, and they answered a questionnaire about the mobile application. During the study, the students answered four weekly questionnaires about the tips and curiosities provided by the mobile application.

In general, most of the teenagers were satisfied with the mobile application’s different functionalities, including physical activity monitoring, tips and curiosities, and questionnaires. By the end of the study, the teenagers indicated that the medical control would be important, and the use of gamification and challenges promoted the use of the mobile application.

The objective of the study was the promotion of healthy nutrition and physical activity habits, and we verified that the habits of some of the teenagers improved. All the available features in the mobile application were positively rated by the teenagers, saying that they would use it in the future. Still, we verified that the design and functionalities of the mobile application need further improvements. Most notably, medical control should be added to increase the motivation of the teenagers. Nevertheless, the study verified that a mobile application could be a complement for the promotion of healthy lifestyles. Due to the pandemic situation, where the teenagers were lacking close contact with others the number of participants in the study was limited. The conclusions could be improved with the distribution of the mobile application with crowdsourcing which would bring more users and more data.

Finally, after the analysis was carried out, it can be concluded that by implementing questionnaires and the use of gamification, it is possible to attract more young people to use mobile applications to promote healthy nutrition, as well as to adopt good physical activity habits.

## Figures and Tables

**Table 1:** Statistical analysis of the points obtained by teenagers with the different questionnaires.

Parameters	Weekly Questionnaire 1	Weekly Questionnaire 2	Weekly Questionnaire 3	Weekly Questionnaire 4
Average	2	1.71	3	2
Standard deviation	0.58	0.95	1.15	1.29
Minimum	1	0	1	0
Maximum	3	3	4	4

**Table 2:** Definition of the points obtained with the answers about the consumption of fruit and vegetables, and sweets, savory snacks, and soft drinks.

Category	Quantity	Points
Servings of fruit and vegetables per day	1 to 2	0
	3 to 5	1
Consumption of sweets, savory snacks and soft drinks per week	Less than three times	1
	Three or more times	0

**Table 3:** Ranking of points obtained by different teenagers.

Points	Number of Teenagers (%)
0	2 (28.6%)
1	3 (42.9%)
2	2 (28.6%)
Total	7 (100.0%)

**Table 4:** Analysis of improvement of diet.

Parameters		Points			Total	*p*-Value
		0	1	2		
Improvement of diet	Kept	1	2	2	5	0.526
	Improved	1	1	0	2	
Total		2	3	2	7	

**Table 5:** Analysis of physical activity habits.

Habits	Level	Physical Activity Level		Total	*p*-Value
		Improved	Kept		
Frequency of physical exercise per week	Less than four times per week	1	3	4	0.486
	Four or more times per week	2	1	3	
Total		3	4	7	
Duration of each session of physical exercise	Less than 1 h	0	1	1	1
	More than 1 h	3	3	6	
Total		3	4	7	

**Table 6:** Satisfaction with the mobile application by age.

Age	Satisfaction with Mobile Application		Total	*p*-Value
	Satisfied	Poor Satisfied		
13–14	3	0	3	1
15–16	3	1	4	
Total	6	1	7	

**Table 7:** Level of motivation with gamification by age.

Age	Level of Motivation with Gamification		Total	*p*-Value
	Motivated	Not Motivated		
13–14	3	0	3	0.429
15–16	2	2	4	
Total	5	2	7	

**Table 8:** Comparison of other studies with the CoviHealth project.

Study	Number of Teenagers in the Starting of the Project	Number of Teenagers at the End of the Project	Number of Weeks of the Study	% Number of Teenagers That Abandoned Along the TIME of the Study	% Number of Teenagers that Abandoned Along the Same Time of CoviHealth Project
Jimoh et al. [43]	34	30	5	11.8	73.1
De Cock et al. [44]	268	55	4	79.5	53.8
Lee et al. [45]	33	21	12	36.4	73.1 *
Spook et al. [46]	30	17	1	43.3	42.3
Reid et al. [47]	29	18	1	37.9	43.3

* CoviHealth project only has five weeks.
